# Role of quercetin and arginine in ameliorating nano zinc oxide-induced nephrotoxicity in rats

**DOI:** 10.1186/1472-6882-12-60

**Published:** 2012-05-02

**Authors:** Laila M Faddah, Nayira A Abdel Baky, Nouf M Al-Rasheed, Nawal M Al-Rasheed, Amal J Fatani, Muhammad Atteya

**Affiliations:** 1 Pharmacology Department, Faculty of Pharmacy, King Saud University, Riyadh, Saudi Arabia; 2 Anatomy Department and Stem Cell Unit, Faculty of Medicine, King Saud University, Riyadh, Saudi Arabia; 3 Department of Pharmacology, Faculty of Pharmacy, King Saud University, P.O. Box. 22452, Riyadh, 11495, Saudi Arabia

**Keywords:** nano- Zinc oxide, Qur, Tumor necrosis factor alpha (TNF-α), Interleukin-6 (IL-6), C-reactive protein (CRP), Arg, Vascular endothelium growth factor (VEGF)

## Abstract

**Background:**

Nanoparticles are small-scale substances (<100 nm) with unique properties. Therefore, nanoparticles pose complex health risk implications. The objective of this study was to detect whether treatment with quercetin (Qur) and/or arginine (Arg) ameliorated nephrotoxicity induced by two different doses of nano zinc oxide (n-ZnO) particles.

**Method:**

ZnO nanoparticles were administered orally in two doses (either 600 mg or 1 g/Kg body weight/day for 5 conscutive days) to Wister albino rats. In order to detect the protective effects of the studied antioxidants against n-ZnO induced nepherotoxicity, different biochemical parameters were investigated. Moreover, histopathological examination of kidney tissue was performed.

**Results:**

Nano zinc oxide-induced nephrotoxicity was confirmed by the elevation in serum inflammatory markers including: tumor necrosis factor alpha (TNF-α), interleukin-6 (IL-6); and C-reactive protein (CRP). Moreover, immunoglobulin (IGg), vascular endothelium growth factor (VEGF), and nitric oxide (NO) were significantly increased in rat serum. Serum urea and creatinine levels were also significantly increased in rats intoxicated with n-ZnO particles compared with the control group. Additionally, a significant decrease in the non-enzymatic antioxidant reduced glutathione (GSH) was shown in kidney tissues and serum glucose levels were increased. These biochemical findings were supported by a histopathological examination of kidney tissues, which showed that in the animals that received a high dose of n-ZnO, numerous kidney glomeruli underwent atrophy and fragmentation. Moreover, the renal tubules showed epithelial desquamation, degeneration and necrosis. Some renal tubules showed casts in their lumina. Severe congestion was also observed in renal interstitium. These effects were dose dependent. Cotreatment of rats with Qur and/or Arg along with n-ZnO significantly improved most of the deviated tested parameters.

**Conclusions:**

The data show that Qur has a beneficial effect against n-ZnO oxidative stress and related vascular complications. Also, its combination with Arg proved to be even more effective in ameliorating nano zinc oxide nephrotoxicity.

## Background

The rapid growth of the nanotechnology industry has led to wide-scale production and application of engineered nanoparticles (NPs). NPs are not only used in industry and medicine but are also increasingly used in various consumer products such as cosmetics, sunscreens, and food products [1]. However, the properties that make them useful are also the cause of concern [2-4]. The ability of NPs to induce toxicity has been attributed to their increased surface reactivity [5,6]. The small particle size of NPs creates a large surface area per unit mass and makes them more reactive in a cell. It has also been proposed that the an increase in the surface area of NPs greatly increase their ability to produce reactive oxygen species (ROS) [7,8]. NPs can enter the human body through different routes such as inhalation, ingestion and injection [9,10]. They may then translocate to blood causing adverse biological reactions in several organs, which are considered to be the secondary major sites of interaction [11,12]. The kidney is particularly susceptible to xenobiotics because of its high blood supply and its ability to concentrate toxins. Alteration of the total glutatahione GSH (tGSH) level in cells can be considered an indication of adaptive response to cell to oxidative damage. Nano-ZnO particles at high concentrations significantly decreased the renal tGSH level compared with control values, which indicates functional damage to kidney tissues.

Considering the hazards of treatment failure, drug resistance and heavy costs associated with current renal therapy, medicinal plants have attracted the interest of many researchers in this field. Qur has been found to protect kidney tissues against age-related NF-κB activity in the kidneys that leads to increased oxidative stress [13]. Moreover, Qur protects the kidneys during ischemia and reperfusion by preserving higher levels of the enzyme xanthine dehydrogenase relative to the injurious enzyme xanthine oxidase [14]. Moreover, it was found that Qur protected the cells against hydrogen peroxide-induced oxidative stress and calcium dysregulation that prevents cell damage [15].

Arginine (Arg) is a nonessential amino acid that has numerous functions in the body*.* Arg plays an important role in cell division, healing of wounds and in immune function [16-18]*.* Oral supplementation of L-Arg has been shown to increase precursors for the synthesis of nitric oxide (NO) [19], reduce the healing time of injuries, [20], and decrease blood pressure [21]. Dietary intervention with L-Arg resulted in amelioration of a number of experimental kidney diseases, such as those caused by subtotal nephrectomy, as well as diabetic nephropathy, [22]. Nitric oxide (NO) synthesis requires Arg, and plays a pivotal role in regulating kidney function in patients with high blood pressure or various renal disorders [23]*.* Impairment of NO production in these vascular epithelial cells is a characteristic feature of heart failure, and it can cause harm to the kidneys.

The objective of this study was to assess renal cell responses to the manufactured NPs to show their potential toxic biological responses and investigate the renoprotective effect of Qur and Arg.

## Methods

### Chemicals

The 50-nm ZnO powder was purchased from Sigma Co. (USA). All other chemicals used in the study were of analytical grade, and were from Sigma and Merck.

### Animals and treatments

Fifty Wistar albino rats (170–200 g) were used. The rats were obtained from the Experimental Animal Care Center, College of Pharmacy, King Saud University. Animals were kept in special cages on a constant 12-h light/12-h dark cycle with air conditioning. Temperature ranged from 20–22°C with 60% humidity. Rats were fed standard rat pellet chow and had free access to tap water ad libitum for one week before the experiment. Animal utilization protocols were performed in accordance with the guidelines provided by the Experimental Animal Laboratory and approved by the Animal Care and Use Committee of King Saud University, College of Pharmacy. After one week acclimation, the rats were kept fasting over night before treatment and were randomly divided into two classes according to the dose of ZnO-nanoparticle that was administered.

Class I consisted of five groups (ten rats per group):

G1: normal healthy animals

G2–G5: animals orally administered 600 mg/kg body weight/day n-ZnO for 5 days [24]*,* and divided as follows:

G2:ZnO-intoxicated animals with a low oral dose (600 mg/kg/day) daily for 5 days.

G3: ZnO-intoxicated animals administered Qur (200 mg/kg) daily [25].

G4: ZnO-intoxicated animals administered Arg (200 mg/kg) [26] daily.

G5: ZnO-intoxicated animals co-administered Arg (200 mg/kg) and Qur (100 mg/kg) daily.

Class II consisted of four groups (G6–G9; ten rats per group) orally administered 1 g/kg body weight/day for 5 days n-ZnO [25] and divided as follows:

G6: ZnO-intoxicated animals with a high oral dose (1 g/kg/day) daily for 5 days.

G7: ZnO-intoxicated animals administered Arg (200 mg/kg) daily.

G8: ZnO-intoxicated animals administered Qur (200 mg/kg) daily.

G9: ZnO-intoxicated animals co-administered Arg (200 mg/kg) and Qur (200 mg/kg) daily.

Qur and/or Arg were orally administered daily for three weeks from the beginning of the experiment. The body weights of rats were recorded before and after the administration period.

At 24 h after the last dose administration, rats were sacrificed by decapitation, and blood was collected. Serum was separated by centrifugation at 3000 rpm. for 10 min and kept at −80°C. Both kidneys were harvested through a midline incision, rinsed in cold isotonic saline, homogenized, and frozen at −80°C for estimations of GSH content. Three kidneys from each group were kept in 10% formalin for histopathological examination.

### Serum biochemical analysis

#### Determination of TNF-α level

The concentration of inflammatory cytokines (TNF-α) in serum was determined using commercially available enzyme-linked immunosorbent assay ELISA kits following the instructions supplied by the manufacturer (DuoSet kits, R&D Systems; Minneapolis, MN, USA).

#### Determination of CRP level

CRP was measured with latex-enhanced immunonephelometry on a Behring BN II Nephelometer (Dade Behring). In this assay, polystyrene beads coated with rat monoclonal antibodies bind CRP present in the serum sample and form aggregates. The intensity of scattered light is proportional to the size of the aggregates and thus concentration of CRP present in the sample. The intra-assay and interassay coefficients of variation for CRP were 3.3% and 3.2%, respectively. The lower detection limit of the assay was 0.15 mg/L [27]*.*

#### Determination of VEGF level

The level of VEGF in serum was determined at 492 nm by quantitative colorimetric sandwich ELISA (R&D systems, UK) in accordance with the manufacturer's instructions [28]. Concentrations were calculated using a standard curve generated with specific standards provided by the manufacturer.

#### Determination of IgG level

The IgG level was measured in serum using a sandwich ELISA. The capture antibody was goat anti-rat IgG (Kirkegaard & Perry Laboratories, Inc., Gaithersburg, MD). Standards were prepared from rat IgG (Sigma Chemical Co., St. Louis, MO). Goat anti-rat IgG peroxidase conjugates were diluted 1:250 in PBS/BSA (from Kirkegaard & Perry Laboratories, Inc.) and used as detecting antibodies. The chromogenic substrate used was 2,2′-azino-di[3-ethyl-benzthiazoline sulfonate] (ABTS; Kirkegaard & Perry Laboratories, Inc.). Color development was detected via optical density at 405 nm using an automated ELISA plate reader (Bio-tek Instruments, Inc., Winooski, VT) and immunoglobulin concentrations were determined by comparison of sample color development to standard curves (Kineticalc, Bio-tek Instruments, Inc.).

#### Determination of IL-6 level

The IL-6 levels were measured by ultra-sensitive ELISA (Quantikine HS Human IL-6 Immunoassay; R&D Systems, Minneapolis, MN) with an analytical CV of 6.3% and a detection level of 0.04 pg/mL [29].

#### Determination of total nitrite/nitrate level

Serum total nitrite/nitrate level concentration (as an indirect measurement of NO synthesis) was assayed using Griess reagent (sulfanilamide and N-1-naphthylethylenediamine dihydrochloride) in acidic medium [30]*.*

#### Determination of glucose level

Glucose level was estimated using the method of Trinder 1969[31]*.*

#### Determination of serum urea, creatinine and uric acid level

Serum samples were assayed for urea, creatinine, and uric acid by using standard diagnostic kits.

### Determination of renal reduced glutathione (GSH) level

Kidney tissue levels of acid-soluble thiols and reduced GSH were determined calorimetrically at 412 nm [32]*.* Homogenates were precipitated with trichloroacetic acid, and after centrifugation, supernatants were used for the estimation of protein thiols (*Protein*-SH) expressed as μmol/mg wet tissue.

### Histopathological techniques

Samples of kidney tissues were collected, and were fixed in a 4% formaldehyde solution for 24 h. They were dehydrated in ascending grades of ethyl alcohol, cleared in xylene, and embedded in paraffin. Paraffin blocks were cut at 4-μm thickness, then fixed on glass slides. Sections were stained with hematoxylin and eosin (H&E) by the following method: they were hydrated using washes with descending grades of alcohol, then hematoxylin was added to stain the nuclei, and afterwards eosin was added to the cytoplasm. Another set of unstained slides were stained with Masson’s trichrome after de-waxing and rehydration of unstained slides. Sections were immersed in Weigert's working hematoxylin for 10 min then washed with water for 5 min, and was rinsed in distilled water. They were then stained with Biebrich scarlet for 5 min followed by rinsing in distilled water. Slides were immersed in phosphotungstic/phosphomolybdic acid for 10 min. The solution was discarded. After that sections were transferred directly into Light Green for 5 min followed by rinsing in distilled water. The sections were immersed in 10.1% acetic acid solution for 1 min to visualize deposition of collagen fibers. This usually makes the sample turn green [33]. The histological changes were estimated semi-quantitatively, as being mild, moderate, or marked by microscopic examination of histological sections.

### Statistical analysis

Data are presented as mean ± S.D. Statistical analysis was performed using an Instat-3 computer program (Graph pad software Inc, San Diego, CA). One way analysis of variance (ANOVA) followed by Bonferroni multiple tests was used to determine the differences between means of different groups. The level of significance was set at *p* ≤ 0.05.

## Results

**Figure 1:**
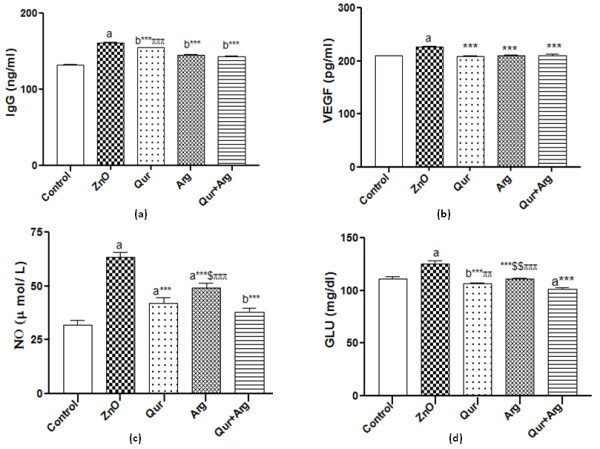
**Effect of Qur and/or Arg treatment on serum IgG (a), VEGF (b), total nitrate/nitrite (c), and glucose (d) concentrations in intoxicated rats with low dose of n-ZnO particles.** Values are expressed as mean ± S*.*D. ^a^*P* < 0.001, ^b^*P* < 0.01 compared with the normal control group, ^***^P < 0.001 compared with the n-ZnO-intoxicated group, ^$$^*P* ≤ 0.01, ^$^*P* ≤ 0.05 compared with the Qur-treated group, ^πππ^*P* ≤ 0.001, ^ππ^*P* ≤ 0.01 compared with the combination group, respectively, using ANOVA followed by Bonferroni as a post-ANOVA test.

**Figure 2:**
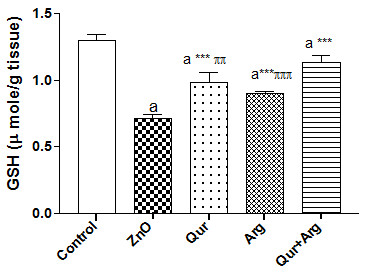
**Effect of Qur and/or Arg treatment on serum IgG (a), VEGF (b), total nitrate/nitrite (c), and glucose (d) concentrations in intoxicated rats with a high dose of n-ZnO particles.** Values are expressed as mean ± S*.*D. ^a^*P* < 0.001 ^b^*P* < 0.01 compared with the normal control group, ^***^P < 0.001 compared with the n-ZnO-intoxicated group, ^$^*P* ≤ 0.01 compared with the arginine-treated group, ^$^*P* ≤ 0.05 compared with the Qur-treated group, ^πππ^*P* ≤ 0.001, ^ππ^*P* ≤ 0.01 compared with the combination group, respectively, using ANOVA followed by Bonferroni as a post-ANOVA test.

**Figure 3:**
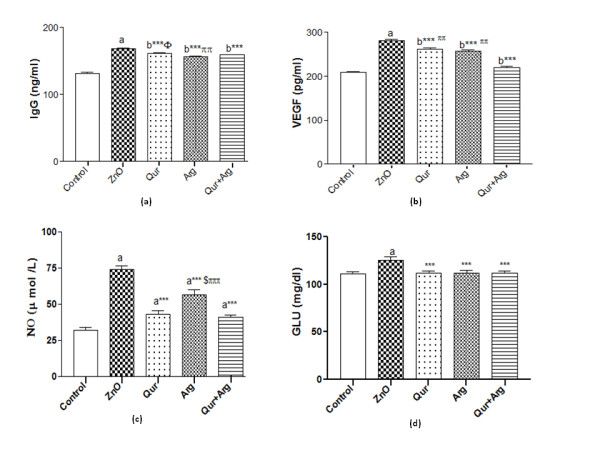
**Effect of Qur and/or Arg treatment on renal glutathione level in intoxicated rats with a low dose of n-ZnO particles.** Values are expressed as mean ± S*.*D. ^a^*P* < 0.001 compared with the normal control group, ^***^P < 0.001 compared with the n-ZnO-intoxicated group, ^πππ^*P* ≤ 0.001, ^ππ^*P* ≤ 0.01 compared with the combination group, using ANOVA followed by Bonferroni as a post-ANOVA test.

**Figure 4:**
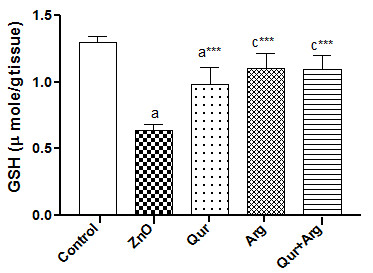
**Effect of Qur and/or Arg treatment on renal glutathione level in intoxicated rats with a high dose of n-ZnO particles.** Values are expressed as mean ± S*.*D. ^a^*P* < 0.001, ^c^*P* < 0.05 compared with the normal control group, ^***^P < 0.001 compared with the n-ZnO-intoxicated group, respectively, using ANOVA followed by Bonferroni as a post-ANOVA test.

The current investigation revealed that the administration of n-ZnO particles, either in low or high doses, significantly elevated (*p* < 0.001) serum inflammatory markers (TNF- α, CRP, and IL-6) levels compared with normal control values (Table 1 &2). Moreover, n-ZnO treatment significantly increased (*p* < 0.001) serum IgG, VEGF, and glucose levels. n-ZnO treatment also increased total nitrite concentration (Figure 1 and Figure 2), with a concomitant decrease in renal GSH content compared with normal control values (*p* < 0.001) (Figure 3 and Figure 4). After estimation of renal function parameters (serum urea, creatinine and uric acid), there were no significant changes in the serum levels of n-ZnO-intoxicated rats as compared with normal control group (*p* < 0.05), with the exception of urea and creatinine levels (Table 3 and 4). Generally, the large dose of n-ZnO induced significant alteration in the previous parameters than that of the low dose. Oral administration of Qur, Arg or a combination of both significantly reduced (*p* < 0.001) serum TNF-α, CRP, IL-6 compared with n-ZnO-intoxicated rats (Table 1 &2). Interestingly, CRP level decreased significantly with Arg treatment. However, the combination of the two agents showed a more powerful effect in alleviating the rise of inflammatory mediator levels. Additionally, serum IgG, VEGF, nitric oxide, and glucose levels decreased significantly (*p* < 0.001), with a concomitant increase in renal GSH content (*p* < 0.001) (Figure 1–4). Arg alone, or in combination with Qur, significantly decreased serum creatinine level as compared with rats intoxicated with high dose of n-ZnO particles (*p* < 0.05) (Table 4 and). There was no significant change either in body weight, kidney weight, or in kidney/body weight % between all the groups (Tables 5 and 6).

**Table 1:** Effect of Qur and/or Arg treatment on serum inflammatory markers level in intoxicated rats with a low dose of n-ZnO particles

**Parameters**	**Control**	**n-ZnO**	**Qur**	**Arg**	**Qur + Arg**
**TNF- α** (pg/ml)	242.7 ± 6.1	361.6 ± 9.2^a^	299.5 ± 3.1^*b # #^	276.12 ± 4.05^*b^	300.6 ± 4.1^*b # #^
**CRP** (ng/ml)	3.16 ± 0.1	4.4 ± 0.20 ^a^	3.4 ± 0.04^*c^	3.3 ± 0.04^*c^	3.1 ± 0.7^* # #^
**IL-6** (pg/ml)	31 ± 2.098	44.33 ± 1.2^a^	42.86 ± 0.49 ^a#^	35.3 ± .81 ^a*^	35.66 ± 1.21 ^a*^

Data are presented as mean ± S.D. from 10 rats, ^*a*^*P* ≤ 0.001, ^*b*^*P* ≤ 0.01, ^*c*^*P* ≤ 0.05 compared with the normal group, ^*^P ≤ 0.001 compared with the n-ZnO-intoxicated group, ^#^*P* ≤ 0.001, ^##^*P* ≤ 0.01 compared with the combination group, respectively, using ANOVA followed by Bonferroni as a post-ANOVA test.

**Table 2:** Effect of Qur and/or Arg treatment on serum inflammatory marker levels in intoxicated rats with a high dose of n-ZnO particles

**Parameters**	**Control**	**n-ZnO**	**Qur**	**Arg**	**Qur + Arg**
**TNF- α** (Pg/ml)	242.7 ± 6.1	371.7 ± 6.1^a^	293.6 ± 4.3^*b^	322.18 ± 1.9^*b#^	314.1 ± 2.9^*b#^
**CRP** (ng/ml)	3.16 ± 0.1	4.6 ± 0.11 ^a^	2.9 ± 0.05^*^	3.0 ± 0.07^*^	3.7 ± 0.055^*b# #^
**IL-6** (ng/ml)	31 ± 2.098	49.57 ± 1.01 ^a^	39.41 ± 0.62^a*^	41.80 ± 0.28a^*#^	44.94 ± 1.02a^*###^

Data are presented as mean ± S.D. for 10 rats, ^*a*^*P* ≤ 0.001, ^*b*^*P* ≤ 0.01 compared with the normal group, ^*^P ≤ 0.001 compared with the n-ZnO intoxicated-group, ^#^*P* ≤ 0.05, ^##^*P* ≤ 0.01, ^###^*P* ≤ 0.01 compared with the combination group, respectively, using ANOVA followed by Bonferroni as a post-ANOVA test.

**Table 3:** Effect of Qur and/or Arg treatment on serum creatinine, urea, and uric acid levels in intoxicated rats with a low dose of n-ZnO particles

**Parameters**	**Control**	**n-ZnO**	**Qur**	**Arg**	**Qur + Arg**
**Creatinine** (mg/dl)	0.4 ± 0.075	0.53 ± 0.077	0.47 ± 0.078	0.44 ± 0.027	0.42 ± 0.069
**Urea** (mg/dl)	21.4 ± 4.88	32.3 ± 6.83 ^a^	28.6 ± 5.47	27.9 ± 2.92	27.2 ± 4.21
**Uric acid** (mg/dl)	2.1 ± 0.55	2.51 ± 0.58	2.44 ± 0.78	2.23 ± 0.21	2.06 ± 0.54

Data are presented as mean ± S.D. for 10 rats, ^*a*^*P* ≤ 0.05 compared with normal group using ANOVA followed by Bonferroni as a post-ANOVA test.

**Table 4:** Effect of Qur and/or Arg treatment on serum creatinine, urea, and uric acid levels in intoxicated rats with a high dose of n-ZnO particles

**Parameters**	**Control**	**n-ZnO**	**Qur**	**Arg**	**Qur + Arg**
**Creatinine** (mg/dl)	0.4 ± 0.075	0.63 ± 0.087 ^a^	0.51 ± 0.066	0.50 ± 0.033 ^*^	0.50 ± 0.045^*^
**Urea** (mg/dl)	21.4 ± 4.88	35.2 ± 5.88 ^b^	29.8 ± 4.57	28.8 ± 2.02	28.2 ± 5.21
**Uric acid** (mg/dl)	2.1 ± 0.55	3.02 ± 0.53	2.84 ± 0.98	2.93 ± 0.61	2.36 ± 0.55

Data are presented as mean ± S.D. from 10 rats, ^*a*^*P* ≤ 0.001, ^*b*^*P* ≤ 0.01 compared with the normal group, ^*^P ≤ 0.05 compared with the n-ZnO-intoxicated group, respectively, using ANOVA followed by Bonferroni as a post-ANOVA test.

**Table 5:** Effect of Qur and/or Arg treatment on body weight, kidney weight, and kidney/body weight % in intoxicated rats with a low dose of n-ZnO particles

	**Body Weight (g)Initial Final**		**Kidney Weight (g)**	**Kidney/Body Weigh %**
**Control**	251.6 ± 9.39	271.6 ± 23.73	1.22 ± 0.164	0.454 ± 0.090
**n-ZnO**	250.4 ± 14.15	280.6 ± 6.50	1.14 ± 0.114	0.405 ± 0.034
**Qur**	242.4 ± 14.84	271 ± 19.30	1.3 ± 0.282	0.482 ± 0.098
**Arg**	238.2 ± 12.27	262.8 ± 19.66	1.22 ± 0.083	0.528 ± 0.074
**Qur + Arg**	235.6 ± 11.19	264.2 ± 17.41	1.18 ± 0.192	0.484 ± 0.081

**Table 6:** Effect of Qur and/or Arg treatment on body weight, kidney weight, and kidney/body weight % in intoxicated rats with a high dose of n-ZnO particles

	**Body Weight (g) Initial Final**		**Kidney Weight (g)**	**Kidney/Body Weigh %**
**Control**	251.6 ± 9.39	271.6 ± 23.73	1.22 ± 0.164	0.454 ± 0.090
**n-ZnO**	232.4 ± 14.87	262.6 ± 22.94	0.94 ± 0.209	0.358 ± 0.051
**Qur**	249.4 ± 22.39	272.8 ± 37.79	1.34 ± 0.243	0.496 ± 0.094
**Arg**	242 ± 19.583	268.6 ± 19.57	1.12 ± 0.211	0.418 ± 0.031
**Qur + Arg**	232 ± 28.87	265 ± 41.085	1.24 ± 0.201	0.492 ± 0.076

**Figure 5:**
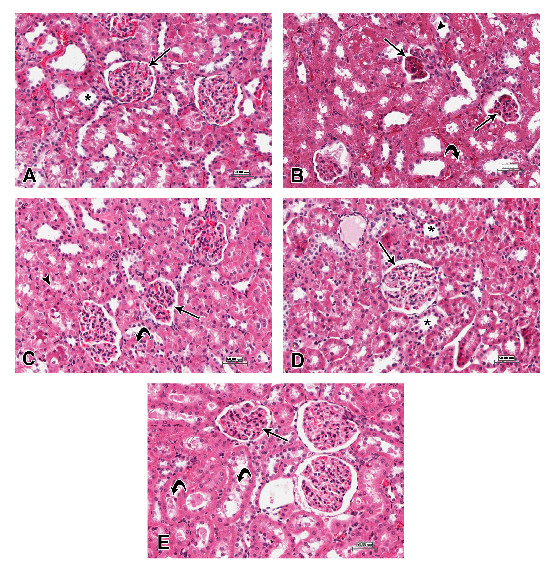
Photomicrographs of kidney sections stained with hematoxylin and eosin (scale bars = 50 μm), showing: (A) control group, normal architecture of renal corpuscles with their glomeruli (arrow) and renal tubules (asterisk); (B) intoxicated group with 600 mg/kg body weight/day n-ZnO particles for 5 days, shrinkage and fragmentation of many glomeruli (arrows) with many renal tubules showing degeneration, necrosis (arrowhead) and exfoliation of their lining epithelium (curved arrow); (C) Qur-treated group, shrinkage and fragmentation in many glomeruli (arrow) with necrosis (arrowhead) and exfoliation of lining epithelial cells of many renal tubules (curved arrow); (D) Arg-treated group, normal architecture of renal corpuscles with their glomeruli (arrow) as well as normal renal tubules (asterisk); and (E) Qur + Arg-treated group, few glomeruli with shrinkage and fragmentation, and few renal tubules with epithelial cell exfoliation (curved arrows).

The results of these biochemical markers were supported by the histopathological examination of kidney tissues stained with HE. This examination revealed that the animals which received a low dose of n-ZnO showed moderate atrophy and fragmentation of many glomeruli compared with control group (Figure 5A). Few renal tubules showed epithelial desquamation, degeneration and necrosis and tubular casts in their lumina. Vascular congestion was also observed in renal interstitium (Figure 5B), but was less than that observed in intoxicated animals with a high dose of n-ZnO. Quercetin treatment showed minimum protective effects on the renal tissues of intoxicated rats (Figure 5C). However, n-ZnO intoxicated animals treated with Arg showed a major improvement in the histopathological destruction observed in kidney tissues. This was shown by minimal glomerular shrinkage and lower rates of tubular epithelial desquamation and degeneration (Figure 5D). Intoxicated animals co-treated with quercetin and Arg showed mild histological changes, in a form of mild glomerular fragmentation and epithelial exfoliation in few renal tubules (Figure 5E).

**Figure 6:**
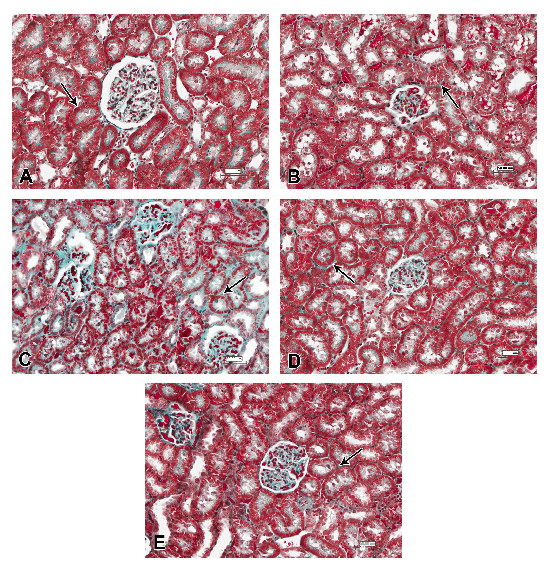
**Photomicrographs of kidney sections stained with hematoxylin and eosin (scale bars = 50 μm), showing: (A) control group, normal renal corpuscles with normal glomeruli (arrow) and renal tubules (asterisks); (B) intoxicated group with 1 g/kg body weight/day for 5 days with n-ZnO particles, shrinkage and fragmentation of many glomeruli (arrows).** In addition many renal tubules show degeneration, necrosis (arrowheads) and exfoliation of their lining epithelium (curved arrow). **(C)** Qur-treated group, many glomeruli reveal shrinkage and fragmentation (arrows) with degeneration, necrosis (arrowheads) and exfoliation of epithelial lining cells of the renal tubules (curved arrow). **(D)** Arg-treated group: Almost all the renal corpuscles with their glomeruli (arrows) as well as the renal tubules (asterisks) are similar to normal architecture. **(E)** Qur + Arg-treated group, there were few renal tubules with degeneration, necrosis (arrowhead) and exfoliation of their lining epithelial cells (curved arrow).

The kidneys of rats treated with a high dose of n-ZnO showed massive atrophy and fragmentation of numerous glomeruli. The renal tubules also showed epithelial desquamation, degeneration and necrosis. Numerous renal tubules showed casts in their lumina. In addition, sever congestion was observed in the renal interstitium (Figure 6B). However, the animals which received n-ZnO and quercetin showed histopathological changes in the kidney similar to those detected in the untreated group (Figure 6C). The animals that received n-ZnO and Arg showed histopathological changes in the kidney similar to those detected in the control group (Figure 6D). However, animals that received n-ZnO, quercetin and arginine showed mild histological changes, with fragmentation of few glomeruli and epithelial exfoliation in few renal tubules (Figure 6E).

**Figure 7:**
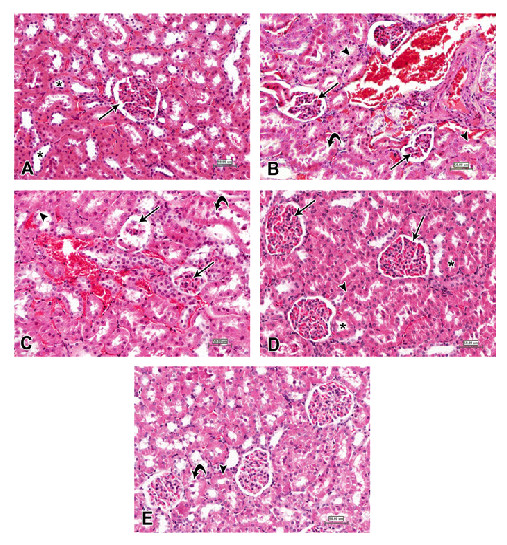
**Photomicrographs of kidney sections from normal and intoxicated rats with 600 mg/kg body weight/day n-ZnO particles for 5 days stained with Masson trichrome (scale bars = 50 μm).** All groups revealed normal distribution of a small amount of collagen fibers (arrow) between the renal tubules in the interstitium.

**Figure 8:**
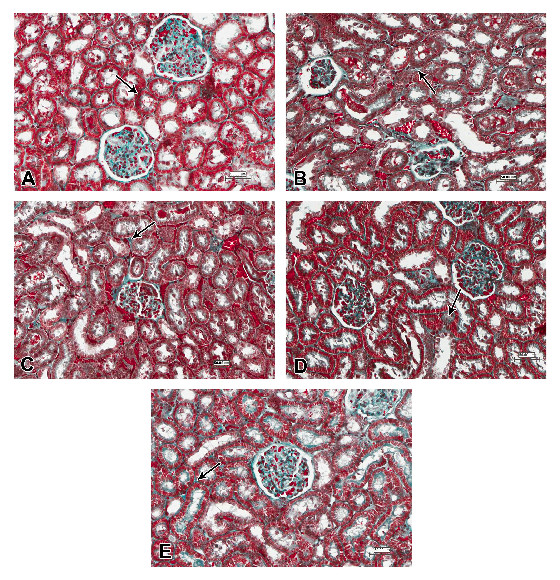
**Photomicrographs of kidney sections stained with Masson trichrome (scale bars = 50 μm), showing the Control group (A) with normal distribution of a small amount of collagen fibers (arrow) between the renal tubules**. **(B)** Intoxicated group with 1 g/kg body weight/day for 5 days with n-ZnO particles. There was an evident increase of deposition of collagen fibers in the interstitium (arrow). **(C)** Qur-treated group, an obvious increase of collagen fibers between the renal tubules (arrow). **(D)** Arg-treated group, there was a small amount of collagen fibers between the renal tubules (arrow). **(E)** Qur + Arg-treated group: Mild increase in collagen fibers between the renal tubules (arrow).

Kidney tissues of animals intoxicated with n-ZnO and stained with Masson’s trichrome showed moderate (with low dose-intoxicated rats) to marked (with high dose-intoxicated rats) increase of collagen fiber deposits in the interstitial tissue (Figure 7B and Figure 8B, respectively) compared with the normal control group (Figure 7A and Figure 8A). Animals that received n-ZnO and quercetin showed a moderate increase of interstitial collagen deposition (Figure 7C and Figure 8C). However, Arg-treated groups showed minimum collagen deposition similar to the control group (Figsure 7D and 8D). Qur- and Arg-treated groups showed a mild increase in collagen deposition (Figure 7E and Figure 8E).

## Discussion

Nanoparticles were found to reach the systemic circulation and disseminate to several organs such as the liver, spleen and kidneys [3,34]. Kidneys play an important role in eliminating xenobiotics from the body and NPs absorbed in the systemic circulation can be excreted by renal clearance [35,36]. Few studies have described the potential toxicity of NPs to renal tubular and glomerular targets [7,37]*.* Kidney damage with morphological, pathological, and cellular changes leading to kidney dysfunction after exposure to NPs has been studied [12,38].

In the present study, low and high doses of ZnO NPs significantly elevated inflammatory cytokine levels, including TNF α-, IL-6, and CRP in rat serum compared with the normal control group. Levels of VEGF, NO and IgG were also significantly elevated. These findings were accompanied with a dose dependent increase in serum urea and creatinine levels in n-ZnO intoxicated rats compared with normal control rats. Tosu et al. [39]*.* elucidated the inflammatory effects of 50- and 100-nm ZnO particles on human umbilical vein endothelial cells (HUVECs). Nano-ZnO concentrations of  ≤ 3 μg/ml resulted in increased cell proliferation, while those of  ≤ 45 μg/ml caused dose-dependent increases in oxidized glutathione levels. Nano-ZnO particles induced a dose-dependent increase in the expression of the intercellular adhesion molecule (ICAM-1) protein, an indicator of vascular endothelium inflammation, and caused marked increases in NF-κB activity. Additionally, TNF-α, a typical inflammatory cytokine, induced ICAM-1 expression in an NF-κB-dependent manner. ZnO also synergistically enhanced TNF-α-induced ICAM-1 expression. Moreover, Fine et al. [40] found that exposures to low concentrations of zinc oxide elevated circulating levels of different cytokines, which could account for the symptoms of the metal fume fever syndrome. This is in accordance with the present study as low and high doses of ZnO NPs significantly elevated cytokines (TNF-α and IL-6) levels in rat serum compared with normal control group.

Growing evidence indicates the beneficial role of a nutrient mixture of L-Arg and Qur in inhibiting the inflammatory response by down-regulating pro-inflammatory cytokine protein expression levels [41]. Moreover, Mostafavi et al. [42] found that Qur and Arg plays a protective role against the imbalance elicited between the production of free radicals and antioxidant defense systems, and suggested that a combination of these two antioxidants may find clinical application where cellular damage is a consequence of reactive oxygen species.

The current study shows that cytokine (TNF-α and IL-6) levels were reduced post-Qur treatment compared with the n-ZnO treated group. This was in accordance with the results of Behling et al. [43] who found that cisplatin-treated rats presented a transitory increase in plasma creatinine levels, tubular cell necrosis and increased immunostaining for vimentin, alpha-SM-actin, fibronectin, and NF-κB in the renal cortex and outer medulla. Meanwhile, Qur treatment attenuated renal functional, histological and immunohistochemical alterations induced by cisplatin [43]. Other studies showed that NF-κB plays a pivotal role in progressive kidney diseases by regulating the accumulation of macrophages [44]. Qur potently inhibited NF-κB activation in cultured rat proximal tubular cells (PTCs), because NF-κB regulates inflammatory signaling and adhesion molecules in PTCs. Additionally, Qur is known to modulate the action of several inflammatory cytokines that are of particular concern to transplant recipients, including IL-1β, IL-2, IL-6, IL-15 and TNF-α [45-47]. Hushmendy et al.[48] found that Qur significantly inhibited cytokine levels and T-cell proliferation, suggesting that it may be effective in reducing transplant rejection. These findings may explain earlier findings that administration of Qur inhibited tubular injury and the elevation in inflammatory cytokine levels.

In the present study Arg significantly reduced the elevated levels of inflammatory cytokines (TNF-α and IL-6) compared with n-ZnO treated group. Moreover, Arg alone or in combination with Qur significantly decreased serum creatinine level as compared with rats intoxicated with a high dose of n-ZnO particles. This was attributed to the fact that Arg has various metabolic and immunologic effects and has been considered to be conditionally essential, particularly under inflammatory and oxidative stress [49]. It has protective effects against oxidative stress and inflammation in different pathological conditions [42]. It can modulate the inflammatory response by modulating the production of inflammatory mediators, such as C-reactive protein as well as cytokine release from activated immune competent cells which play a crucial role in the progression of the pathology [50]*.*

Elevation of CRP level post_n-ZnO administration compared with the normal control group in the current work was in accordance with the work of **Kim et al.**[27]*,* who clarify that CRP is related to the incidence of many pathological condition as coronary heart disease, hypertension, and inflammation. Both Qur and/or Arg treatments reduced CRP level compared with n-ZnO intoxicated group. This is in agreement with **Kleemann et al.**[51] who clarified that dietary intake of flavonoids such as Qur was associated with lower CRP levels. Moreover, **Wu et al.**[50] found that Arg can modulate inflammatory response by modulating the production of inflammatory mediators, such as C-reactive protein*.*

The increase in the circulating IgG in rat serum intoxicated with both doses of ZnO-NPs is an immune response induced by NPs toxicity. It is suggested that the increase in the circulating antibodies are the result of the production of different inflammatory cytokines, including TNF-α, with potential impact on immunoglobulin production during inflammation. These results may indicate tha tZnO-NPs induced inflammatory kidney injury through production of the inflammatory mediators [52]*.* The IgG level was reduced by Arg and Qur treatments. This may explain the role of these agents to suppress the release of inflammatory mediators.

There was a significant increase in serum nitrite/nitrate level in n-ZnO intoxicated rats. High amounts of NO are released from the inducible NO synthase (iNOS) isoform in response to inflammatory stimuli from a variety of cell types [53,54]*.* Renal proximal tubule and inner medullary collecting duct cells can produce NO via expression of an inducible isoform of nitric oxide synthase [55]. Mesangial cells and invading immune cells are capable of expressing iNOS upon stimulation with TNF-α and IL-1b. The release of large amounts of NO in the glomerulus may lead to the progression of renal failure during several forms of glomerulonephritis [56]. Increased tissue factor expression is thought to play a significant role in the development of multiorgan system failure in acute injury [57]. Vascular endothelial growth factor (VEGF), which is a potent mitogen for endothelial cells, has been reported to be expressed in several tissues, including the kidney. The expression of various tissue factors, cytokines, and chemokines in response to inflammatory tissue injury stimulates VEGF synthesizing cells such as platelets, immune cells, and inflammatory cells [58-60]. Besides its mitogenic properties, VEGF is able to promote angiogenesis and increase vascular leakage [61,62]. However, it was found that stimulation of angiogenesis may contribute to the transition from acute to chronic inflammation. Some investigations have revealed that new vessels can significantly contribute to perpetuation of the inflammatory response by expressing chemokines and adhesion molecules promoting the recruitment of inflammatory cells [63]. This suggests the possibility that TNF-α and VEGF might act synergistically to potentiate kidney injury and/or systemic organ dysfunction [64]*,* and explain the elevation in TNF-α, NO, and VEGF levels post-n-ZnO treatment compared with the control group.

The use of anti-angiogenic agents may then represent an attractive alternative therapeutic tool to prevent or significantly reduce fibrosis progression .The protective ingestion of the agents used alone or in combination, markedly reduced the dramatic increase in this angiogenic biomarker in sera of n-ZnO intoxicated rats, suggesting their anti-angiogenic beneficial action. Previous investigations revealed the role of Arg in reducing VEGF expression [65].

Serum nitrite/nitrate was significantly decreased upon treatment of intoxicated animals with Qur or with the Qur and Arg combination but not with Arg alone. However, the increase in NO level in the Arg-treated group was accompanied by a significant increase in renal GSH content of n-ZnO intoxicated rats. The n-ZnO treated group showed a significant increase in free radical generation after n-ZnO treatment with resultant damaging effect on the kidney tissue as evidenced from histopathological examination (Figure 5,8). Data of Arg-treated group suggests that pharmacological increases in NO levels did not exacerbate the increase in free radical formation. High levels of NO or Arg in the Arg-treated group may be protective, probably due to their property of scavenging free radicals as well as inhibiting xanthenes oxidase (XO) enzyme [66]*.*

In the present study, GSH levels were significantly reduced after n-ZnO treatment, while treatment with both Arg and Qur increased renal GSH content when compared with the n-ZnO treated group. This was attributed to the antioxidant properties of both agents. The Qur antioxidant effect is based on its ability to quench hydrogen peroxide [14]. Qur reduces platelet aggregation and adhesion by reducing hydrogen peroxide production [14]. Qur attenuated cyclosporine-induced oxidative stress by restoring the activity of the antioxidative enzymes glutathione peroxidase and catalase, preventing its nephrotoxic action and kidney damage [67]. Arg significantly reduced lipid peroxidation and increased GSH content in the heart tissue of exhaustively exercised rats [68]*.* These findings augment the current results in which the combination of both agents significantly decreased kidney damage by synergistically increasing cellular levels of GSH.

These biochemical findings were supported by the histopathological examination of kidney tissues, which clarified that animals received either low or high doses of n-ZnO particles showed moderate to massive atrophy and fragmentation of numerous glomeruli. The renal tubules showed epithelial desquamation, degeneration and necrosis. Some renal tubules showed casts in their lumina. Sever congestion was also observed in renal interstitium. These hazardous effects were dose-dependent. Co-treatment of n-ZnO_intoxicated rats with Qur or Arg, significantly improved most of the deviated histopathological parameters. The combination of Qur and Arg nearly prevented the damaging effect of n-ZnO intoxication that was manifested microscopically by extensive improvements in the histological features of kidney cells in the form of fragmentation of few glomeruli and epithelial exfoliation in few renal tubules as well as marked reduction in deposition of collagen fibers in the interstitial tissues.

## Conclusion

In this study, the ability of ZnO-NPs to exert different cytotoxic effects was demonstrated. This was evidenced from the elevation in inflammatory cytokines (TNF, IL6, and CRP), IgG, VEGF, creatinine levels, as well as NO induction. These altered parameters contribute to nephrotoxic potential of n-ZnO particles which was clear from the histopathological examination. Treatment with either Arg, Qur, or their combination successively alleviated the alterations in the previous biomarkers, as well as effectively reducing the histopathological changes in n-ZnO cytotoxic rats. This may be related to their ability to attenuate the extent of liberation of ROS and inflammatory cytokines induced by such nanoparticles.

## Competing interests

The authors declare that they have no competing interests.

## Authors’ contributions

LF: participated in the design of the study, participated in the experimental part of the study, evaluated the therapeutic effect of the used antioxidants on different biological function. NA: carried out the experimental part of the study, participated in the design of the study, performed the statistical analysis, drafted the manuscript. NR: conceived the study, and participated in its design and coordination. NR: participated in manuscript design and coordination. AF: participated in manuscript design and coordination. MA: participated in the histopatholigical part. All authors read and approved the final manuscript.

## Pre-publication history

The pre-publication history for this paper can be accessed here:

http://www.biomedcentral.com/1472-6882/12/60/prepub
