# Erratum to: Role of quercetin and arginine in ameliorating nano zinc oxide-induced nephrotoxicity in rats

**DOI:** 10.1186/s12906-016-1291-x

**Published:** 2016-08-26

**Authors:** Laila M. Faddah, Nayira A. Abdel Baky, Nouf M. Al-Rasheed, Nawal M. Al-Rasheed, Amal J. Fatani, Muhammad Atteya

**Affiliations:** 1 Pharmacology Department, Faculty of Pharmacy, King Saud University, Riyadh, Saudi Arabia; 2 Anatomy Department and Stem Cell Unit, Faculty of Medicine, King Saud University, Riyadh, Saudi Arabia; 3 Department of Pharmacology, Faculty of Pharmacy, Al-Azhar University, Cairo, Egypt

## Erratum

Following publication of the original article [1] it was brought to our attention that affiliation 1 and 3 referred to the same institution, despite the fact that these should be two distinct affiliations. Please therefore note that the correct version of affiliation 3 should read as follows:

^3^Department of Pharmacology, Faculty of Pharmacy, Al-Azhar University, Cairo, Egypt
